# Supplementary material for: MetaRibo-Seq measures translation in microbiomes
Source: Nat Commun. 2020 Jun 29;11:3268. doi: 10.1038/s41467-020-17081-z (PMC7324362; doi:10.1038/s41467-020-17081-z)
Supplement: Supplementary file 10 — Supplementary Data 7 [file 41467_2020_17081_MOESM10_ESM.zip › File2/Confidence_High_Taxonomy/319931_out.krona.html]

Javascript must be enabled to view this page.

members
magnitude
magnitudeUnassigned
count
unassigned
taxon
rank

319931\_out

44

2
superkingdom
44

phylum
44
1239

186801
class
44

order
44
186802

186806
1
family

genus
1
1730

39485
1
species

SRS146812\_contig\_number\_42699

541000
family
1

species
1
1898205

SRS012849\_contig\_number\_12224

186803
family
8

33042
5
genus
2

SRS011302\_contig\_number\_contig-100\_1397.128914SRS013476\_contig\_number\_1848


SRS011084\_contig\_number\_29601SRS012969\_contig\_number\_14799
33043
2
species


SRS012273\_contig\_number\_38819
2293091
1
species

species
1
39491

SRS142890\_contig\_number\_30758

1898203
2
species

SRS015065\_contig\_number\_14579SRS024132\_contig\_number\_48684

family
34
31979

34
genus
3

SRS013940\_contig\_number\_15226SRS063518\_contig\_number\_15532SRS147614\_contig\_number\_7224
1485

2293018
species
3

SRS017307\_contig\_number\_22957SRS017859\_contig\_number\_4742SRS1041134\_contig\_number\_15144

2293049
12
species

SRS013098\_contig\_number\_47359SRS015782\_contig\_number\_19384SRS016335\_contig\_number\_22370SRS024663\_contig\_number\_12634SRS047044\_contig\_number\_5032SRS049995\_contig\_number\_3103SRS077194\_contig\_number\_24061SRS1041137\_contig\_number\_28475SRS144135\_contig\_number\_24815SRS144506\_contig\_number\_34845SRS147766\_contig\_number\_9732SRS893383\_contig\_number\_36019


SRS018836\_contig\_number\_3125SRS020233\_contig\_number\_42800SRS044535\_contig\_number\_16415SRS050752\_contig\_number\_17402SRS076804\_contig\_number\_12190SRS1041039\_contig\_number\_20643SRS1041145\_contig\_number\_14330SRS104311\_contig\_number\_17688SRS148196\_contig\_number\_41983
species
9
1262774

2293031
3
species

SRS011239\_contig\_number\_22480SRS048060\_contig\_number\_9430SRS051031\_contig\_number\_14186


SRS012969\_contig\_number\_37387SRS014235\_contig\_number\_19181SRS075773\_contig\_number\_27910SRS143895\_contig\_number\_18241
2293006
4
species
